# Identifying predictors of use of mental health services and supports during the COVID-19 pandemic: an analysis of the CHILD COVID-19 Add-on Study

**DOI:** 10.3389/frhs.2026.1780564

**Published:** 2026-06-18

**Authors:** Scott James Ramsay, Hunter Lloyd, Manon Ranger, Stuart Turvey, Elinor Simons, Larisa Lotoski, Theo Moraes, Leslie E. Roos, Padmaja Subbarao, Meghan B. Azad

**Affiliations:** 1 School of Nursing, University of British Columbia, Vancouver, BC, Canada; 2 BC Children's Hospital Research Institute, Vancouver, BC, Canada; 3 Djavad Mowafaghian Centre for Brain Health, Vancouver, BC, Canada; 4 Department of General Pediatrics, BC Children's Hospital, Vancouver, BC, Canada; 5 Children's Hospital Research Institute of Manitoba, University of Manitoba, Winnipeg, MB, Canada; 6 Department of Pediatrics and Child Health, University of Manitoba, Winnipeg, MB, Canada; 7 Department of Pediatrics and Program in Translational Medicine, SickKids Research Institute, Toronto, ON, Canada; 8 Department of Psychology, University of Manitoba, Winnipeg, MB, Canada; 9 Department of Medicine, McMaster University, Hamilton, ON, Canada

**Keywords:** children, COVID-19 pandemic, mental health services and supports, pediatric, survey

## Abstract

**Objective(s):**

The aim of this work was to explore the rate of and factors associated with the use of mental health services and supports (MHSS) in a Canadian cohort of children and adolescents during the coronavirus disease 2019 (COVID-19) pandemic.

**Study design:**

We analyzed data from 1,554 parent–child dyads participating in the CHILD COVID-19 Add-on Study, surveyed between January 2021 and March 2022.

**Results:**

The overall rate of self-reported access to MHSS over 15 months was 17% among 1,100 participants. Bivariate analyses identified multiple predictors associated with MHSS use. After adjustments in the logistic regression models, the following factors were significantly associated with MHSS use: accessing COVID-19-related income support [adjusted odds ratio (aOR), 0.56; 95% confidence interval (CI), 0.35–0.89]; missed school (aOR, 1.74; 95% CI 1.16–2.61); being slightly worried (aOR, 1.86; 95% CI 1.15–3.00) or very/extremely worried (aOR, 5.25; 95% CI 2.38–11.56) in the past 2 weeks compared with not worried; and having a chronic health condition (aOR, 2.78; 95% CI, 1.85–4.19). In contrast, having a mental health rating of very good (aOR, 0.45; 95% CI, 0.28–0.73; *p* < 0.01) or excellent (aOR, 0.30; 95% CI 0.17–0.52; *p* < 0.001) compared with good before COVID-19 was protective against MHSS use. Subanalysis comparting participants with a chronic health condition found similar effect sizes for being very/extremely worried (aOR, 5.67 vs. aOR 4.98) compared with not worried in the past 2 weeks, in relation to MHSS use.

**Conclusion(s):**

Mental health service use was notable during COVID-19, particularly among children with chronic health conditions. Further research on the use of MHSS is needed to provide effective mental healthcare for children.

## Introduction

Mental health challenges are a growing concern among children. The coronavirus disease 2019 (COVID-19) pandemic had wide-ranging effects on mental health across the lifespan, including heightened anxiety and depression, physical health complaints, sleep pattern disturbances, development of phobias, cognitive functioning issues, and trauma responses (1, 2). In youth, there was a significant impact on mental health during COVID-19: Approximately one in five Canadian youths were affected by a mental health illness or disorder, with only 20% receiving appropriate treatment or mental health services (2). Given that childhood is a critical period that shapes mental health across the lifespan, these concerning trends highlight the urgent need to improve screening, enhance utilization of mental health resources, and implement targeted interventions that support the wellbeing of children and adolescents.

The impact of the COVID-19 pandemic was far-reaching and unprecedented, particularly on healthcare systems. While use of most health services and supports decreased during the COVID-19 pandemic, use of mental health services and supports (MHSS) increased compared with prepandemic levels (3). This finding is especially true for virtual, online, and telehealth options, as lockdown measures limited the availability of in-person MHSS (4). These rapid adaptations in MHSS delivery highlight the resilience and innovativeness of healthcare systems in responding to crisis. However, further research is needed to evaluate MHSS use across diverse population segments.

In studies focused on children and adolescents, researchers have observed that those most directly impacted by COVID-19 (including pediatric patients, their families, and healthcare providers) with pre-existing mental health conditions experienced greater challenges in MHSS use (5, 6). However, there is insufficient insight into how those with other pre-existing health conditions used MHSS during the pandemic. It is well established that children and adolescents with chronic health conditions oftentimes utilize mental health services, but it is not known how the COVID-19 pandemic impacted utilization (7). In addition, few studies have examined factors affecting young people's ability to access MHSS in Canada (8). This critical knowledge gap limits our ability to develop effective strategies to address inequities in mental healthcare use among children and adolescents.

The main objectives of this study were as follows: (1) to describe the characteristics of Canadian children and adolescents surveyed between January 2021 and March 2022 in a national cohort, and report their rate of mental health service use; (2) to identify the factors associated with mental health service use during COVID-19; and (3) to investigate how those with pre-existing health conditions used MHSS during the pandemic. We analyzed data from a national cohort study, which included a large dataset from child and family dyads (9). We examined whether children and adolescents with a pre-existing chronic health condition, similar to those with a mental health condition, were more likely to use mental health services during the COVID-19 pandemic. In addition, consistent with previously published work (10), we hypothesized that a higher prepandemic mental health rating would be protective—that is, children and adolescents with a higher self-reported rating of their own mental health would be less likely to use services and supports compared with children and adolescents with a poorer mental health history.

## Methods

### Study design and population

This analysis presents data collected as part of the descriptive, cross-sectional CHILD COVID-19 Add-on Study (10). This is a prospective longitudinal study embedded within the existing CHILD cohort (9), which has followed families with children born in British Columbia, Alberta, Manitoba, and Ontario between 2009 and 2012 (90.7% retention to date). CHILD households (including index children, siblings and parents) were surveyed between January 2021 and March 2022 to examine the impact of the COVID-19 pandemic on children and parents living in the same household. Further details on cohort profile are available in the published CHILD COVID-19 Add-on Study (10).

**Figure 1:**
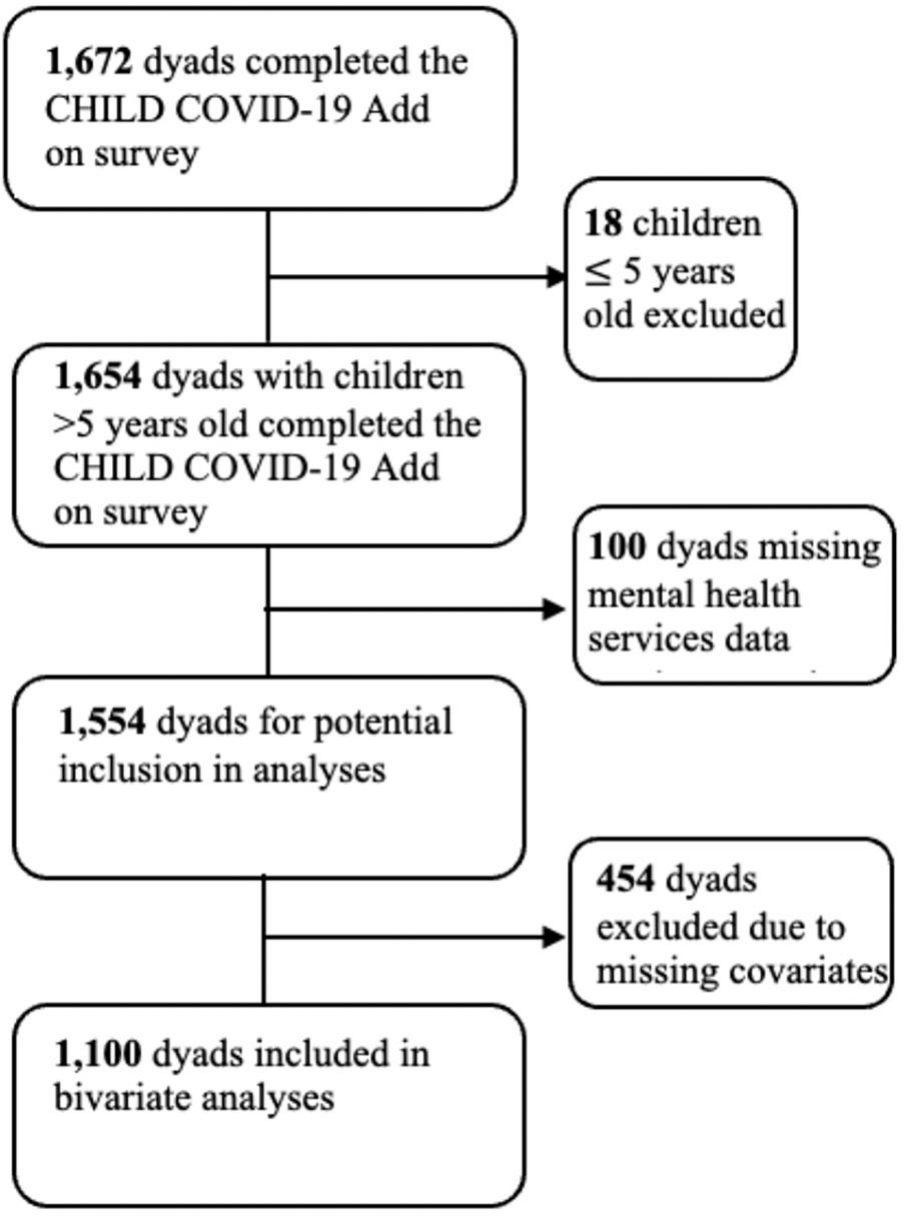
Participant recruitment for the CHILD COVID-19 Add-on Study using the participant pool from the original CHILD cohort study (9).

The sample for the present study included matched parent–child dyads. A total of 1,672 children and adolescents (aged 0–18 years) had completed questionnaires in the CHILD COVID-19 Add-On Study with a matched parent questionnaire (81% response rate). Of these, 18 children were <6 years old and were excluded, given our focus on self-reported mental health status. In addition, 99 surveys had missing data for the outcome variables and were excluded, leaving 1,554 parent–child dyad surveys for potential inclusion in the study. Before bivariate analysis, the amount of missing data was assessed for each predictor variable. If there was a significant amount of missing data (>30%) for a particular predictor variable, that variable was excluded from the dataset. A total of 454 dyads were excluded from analyses after variable exclusion, leaving 1,100 dyads in the study for statistical analyses (Figure 1).

### Questionnaires

The questionnaires used in the Add-on Study (10) were adapted from other Canadian COVID-19 studies and addressed the following topics: individual demographic characteristics, employment status, COVID-19 pandemic government support use, mental and physical health, school closures, adherence to non-pharmaceutical public health measures, screen time and use of technology for school or other purposes, COVID-19 history, and vaccination. Three versions of the questionnaire were distributed, designed for completion by children (about themselves), adults (about themselves), or parents/caregivers (about their children). Adults able to provide consent completed the adult questionnaire. All remaining participants (i.e., children enrolled through assent or caregiver consent) were asked to complete the child questionnaire. This study primarily focused on the surveys completed by children, with supporting documentation from the parent/caregiver survey about their child.

### Definitions and measures

The CHILD COVID-19 questionnaire consisted of five domains with identical content across versions, tailored by wording for respondent type (e.g., parent or child). In the healthcare access and pandemic-driven lifestyle changes domain, six questions asked about use of common MHSS, including individual therapy, group therapy, family therapy, a mental health mobile app in general, a mental health mobile app to interact with a therapist, or a crisis helpline. The response options were “yes,” “no,” “no but would be interested,” or “no response.” To examine use of MHSS in general, the child's responses to the six questions were recorded into a new binary variable called “mental health services use”; a response of yes to any of the questions was coded as yes, while a response of “no” or “no but would be interested” was coded as no given that participants had not used MHSS.

Chronic health conditions were defined using the Chronic Diseases list from the Public Health Agency of Canada (PHAC) (11). Chronic diseases are characterized as long-lasting (3+ months) with no cure (11). The parent/caregiver questionnaire included the extra domain of medical history, which asked about the presence of health conditions, past or current. Of these, 15 conditions are on the PHAC Chronic Diseases list (11), such as arthritis, cancer, heart disease, epilepsy, asthma, diabetes, attention-deficit disorder, anxiety, and depression (Supplementary Table 1). Responses to those questions were used to generate a new binary variable, where the parent responded “yes” to the child having any of the listed chronic health conditions, past or present, or “no,” indicating the child had not.

A single question regarding the child's mental health rating prior to COVID-19 was also asked (“What was your overall mental health rating before COVID-19?”) and ranked ordinally from 1 = Excellent to 4 = Fair/Poor. There was a question on being worried about mental health in the past 2 weeks [“During the past 2 weeks, how worried have you been about your mental/emotional health (how you think and feel) being influenced by COVID-19?”]. Responses were ranked ordinally from 1 = Not at all to 4 = Very/Extremely. There was a question about the number of hours the child slept per night in the previous 2 weeks. We also examined responses to questions about whether the child had received a positive COVID-19 test and whether the family had accessed COVID-19-related income support. Child sociodemographic data in the study included age, geographic location, gender, and ancestry.

### Statistical analyses

All analyses were performed using IBM SPSS Statistics (version 30.0). Data were deidentified as described by the original CHILD Study (9) and assigned unique participant IDs, linking each child with their parent. The parent and child datasets were matched and merged, as our study objective required information from both surveys. Only cases that had responded to all questions of interest were included in analyses, and incomplete cases were excluded by list-wise deletion (12). Before conducting analyses to address the research objectives, descriptive statistics were conducted to document the demographics and health profiles of children who did or did not use mental health services during the pandemic. Categorical variables were summarized using frequencies and percentages. The mean and standard deviation were calculated for normally distributed continuous data; the median and range were calculated for skewed data.

To identify the predictors of child or adolescent MHSS use, a series of analyses were conducted. Chi-square tests were used to identify variables to include in the binary logistic regression. A hierarchical series of binary logistic regressions were performed nested by household to predict variables within dyads associated with use of MHSS. Model 1 included demographic covariates of age, gender, and family access to COVID-19 income support. Model 2 added covariates related to participants' mental health, including participants' self-reported mental health rating pre-COVID-19, number of hours of sleep, and the number of school days missed. Model 3 added variables addressing whether the participant had a current or prior diagnosis of a chronic health condition. Model 4 included interactions between the chronic health condition variable and all variables added in Model 2. Finally, a subanalysis was conducted by chronic health condition to differentiate predictors between children with and without chronic health diagnosis.

A Box–Tidwell transformation test was completed for the two continuous variables, age and number of days of missed school (13). The interaction terms were not significant, indicating that no further transformation of either variable was required for the binary logistic regression.

### Ethical considerations

All eligible participants for inclusion were recruited during in-person clinical visits for the CHILD Cohort Study (9) or via remote communication channels (telephone, video conferencing, or electronic mail) between November 2020 and May 2021 (10) (Figure 1); further details on consent procedures are available in the published protocol (10). We obtained ethical approval for this study from the University of British Columbia Research Ethics Board (ethics approval number H24-01336).

## Results

**Figure 2:**
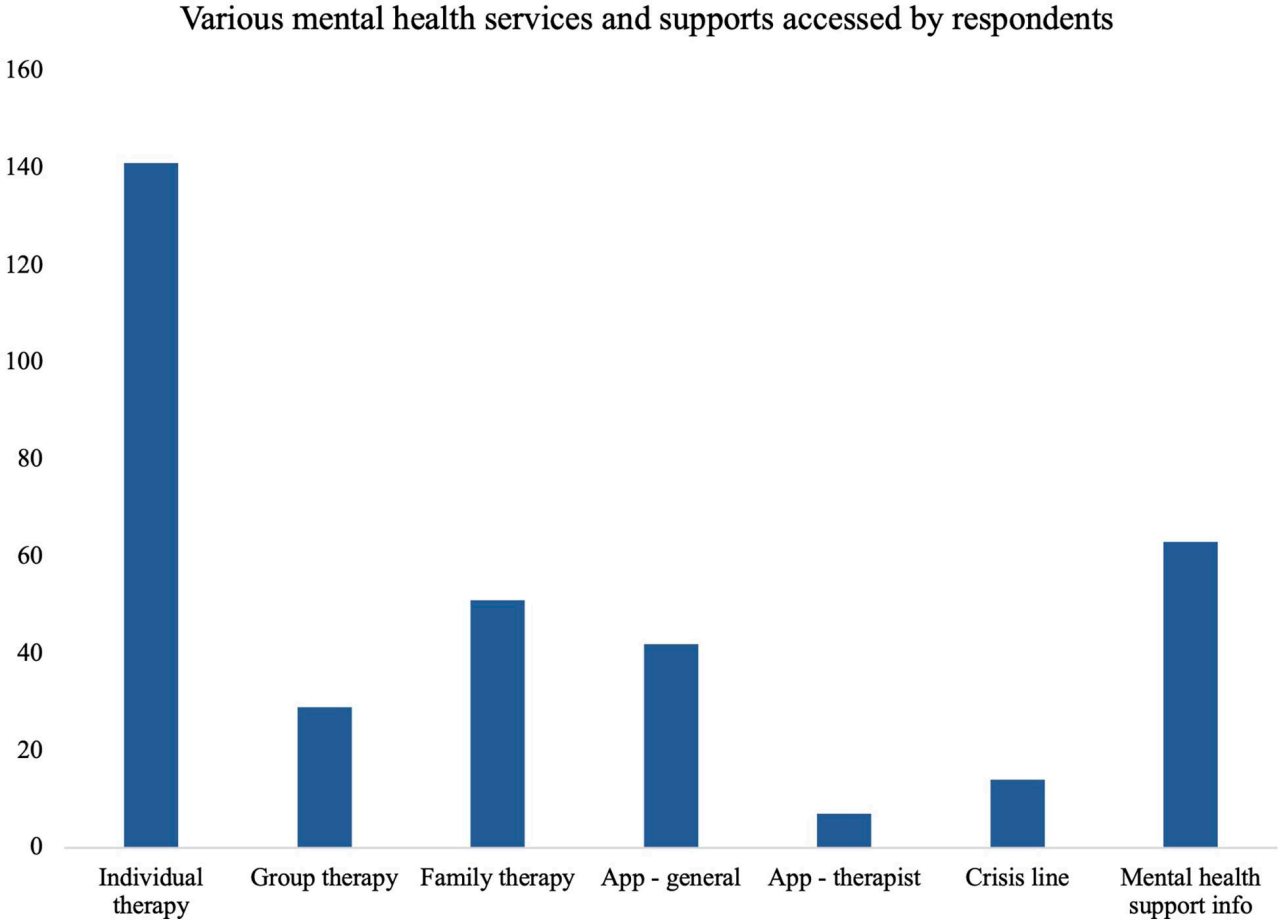
Frequency count of participants who self-reported having accessed a variety of mental health services and supports (*N* = 100).

The demographics of the participants included in this study are listed in Table 1 (*N* = 1,100). Approximately 52% of participants identified as male, with a mean age of 10.3 (SD = 1.9) years (range 6–18 years old). Ancestry was reported as European for 57.8% of participants, 25.5% identified with mixed ancestry, 6.3% self-identified as Indigenous, and 2.7% reported unknown or preferred not to answer. Notably, 26.9% of the sample had a current or past chronic health condition (see Supplementary Table 1 for list of conditions). With respect to MHSS use, we found that 17% of the cohort reported using one or more of the following between January 2021 and March 2022: individual therapy, group therapy, family therapy, a mental health mobile app, a mental health mobile app to interact with a therapist, or a crisis helpline (Figure 2).

**Table 1:** Demographics of sample (*N* = 1,554 children–parent dyads from the CHILD COVID-19 Add-on Study, surveyed between January 2021 and March 2022).

Characteristic	Descriptive	Respondents	Non-respondents
		*N* = 1,100	*N* = 454
		*n* (%)	*n* (%)
Age (years)	Mean (standard deviation)	10.3 (1.9)	10.3 (2.1)
Sex	Male	574 (52.2)	234 (51.5)
	Female	518 (47.1)	213 (46.9)
	Prefer not to answer	8 (0.7)	7 (1.6)
Geographic location^a^	British Columbia	214 (19.9)	96 (20.9)
	Alberta	356 (32.7)	103 (22.4)
	Manitoba	282 (26.3)	156 (34.0)
	Ontario	212 (19.5)	97 (21.1)
	Other province/territory	10 (0.9)	7 (1.5)
Ancestry^a^	European	619 (57.8)	233 (50.1)
	Asian	83 (7.6)	24 (5.2)
	North American, not of Indigenous descent	11 (1.0)	18 (3.9)
	Indigenous	68 (6.3)	43 (9.2)
	Mixed ethnicity	277 (25.5)	118 (25.4)
	Unknown/prefer not to answer	30 (2.7)	29 (6.3)
Accessed COVID-19 income support^b^	Yes	335 (38.8)	186 (40.0)
	No	752 (69.2)	279 (60.0)
Average amount of sleep per night during the week (h)^a^	<6	14 (1.3)	5 (1.1)
	6–8	167 (15.6)	62 (14.0)
	8–10	779 (71.5)	319 (71.8)
	>10	129 (11.8)	58 (12.5)
Positive COVID-19 test	Yes	16 (1.5)	7 (1.5)
	No	1,073 (98.5)	458 (98.5)
Mental health score^b^^,^^f^	Excellent	377 (34.6)	170 (36.7)
	Very good	441 (40.5)	184 (39.7)
	Good	224 (20.6)	88 (19.0)
	Fair/poor	47 (4.3)	21 (4.5)
Worried about mental/emotional health^c^	Not at all	557 (67.6)	20 (80.0)
	Slightly	174 (20.4)	5 (20.0)
	Moderately	65 (7.6)	
	Very/esxtremely	38 (4.4)	
Chronic health condition diagnosis^e^	Yes	293 (26.9)	133 (28.6)
	No	796 (73.1)	332 (71.4)
School missed of current school year^d^	Median ≤	553 (51.8)^f^	241 (54.5)
	>Median	515 (48.2)^f^	201 (45.5)

a *N* = 1,553.

b *N* = 1,552.

c *N* = 879.

d *N* = 1,510.

e Arthritis, cancer, heart disease, hypertension, stroke, asthma, insulin-dependent and independent diabetes, inflammatory bowel disease, attention-deficit disorder, bipolar disorder, anxiety, and depression.

f Median = 2; range = 0–100.

### Bivariate analysis

Before running the regression analyses, bivariate analyses were conducted to identify potential predictor variables. Table 2 presents the results of chi-square tests examining associations between the binary MHSS use variable and potential predictors of interest. Participants using MHSS were more likely to be female, be part of families that used COVID-19 income support, have fair or good previous mental health ratings, sleep <8 h per night, have missed >2 school days during the academic year, and have a current or prior chronic health condition.

**Table 2:** Chi-square test result predicting using mental health support(s) by demographic characteristics and health factors (*N* = 1,100).

Characteristic	Used mental health services	Did not use mental health services	*p*-Value
	*n* (%)	*n* (%)	
	187 (17%)	913 (83%)	
Age	187 (17)	913 (83)	0.11^a^
Geographic location^a^			0.132
British Columbia	38 (17.4)	180 (82.6)	
Alberta	73 (20.4)	285 (79.6)	
Manitoba	44 (15.4)	241 (84.6)	
Ontario	27 (12.7)	186 (87.3)	
Other province/territory	3 (27.3)	8 (72.7)	
Sex			0.044*
Male	85 (14.8)	489 (85.2)	
Female	102 (19.7)	416 (80.3)	
Prefer not to answer	0 (0.0)	8 (100.0)	
Ancestry			0.186
European	114 (18.2)	513 (81.8)	
Asian	10 (12.1)	73 (87.9)	
North American	1 (9.1)	10 (91.9)	
Indigenous	16 (23.2)	53 (76.8)	
Mixed ethnicity	45 (16.2)	233 (83.8)	
Other	0 (0.0)	7 (100)	
Unknown/prefer not to answer	1 (4.2)	23 (95.8)	
Accessed COVID-19 income support			0.044*
Yes	46 (13.6)	292 (86.4)	
No	141 (18.6)	619 (81.4)	
Average amount of sleep per night during the week (h)			0.006**
<6	6 (42.9)	8 (57.1)	
6–8	38 (22.8)	129 (77.2)	
8–10	119 (15.3)	661 (84.7)	
>10	20 (15.5)	109 (84.5)	
Positive COVID-19 test			0.249
Yes	1 (6.3)	15 (93.7)	
No	186 (17.2)	898 (82.8)	
Pre-COVID mental health score			<0.001**
Excellent	37 (9.8)	342 (90.2)	
Very good	68 (15.2)	379 (84.8)	
Good	63 (28.0)	162 (82.0)	
Fair/poor	18 (37.5)	30 (62.5)	
How worried have you been about your mental health in the last 2 weeks?			<0.001**
Not at all	69 (11.8)	515 (88.2)	
Slightly	41 (23.6)	133 (76.4)	
Moderately	18 (27.7)	47 (72.3)	
Very/extremely	17 (44.7)	21 (55.3)	
Chronic health condition diagnosis^b^			<0.001**
Yes	90 (30.7)	203 (69.3)	
No	97 (12.0)	710 (88.0)	
Number of missed school days^c^			<0.02*
≤Median	81 (14.5)	478 (85.5)	
>Median	103 (19.8)	417 (80.2)	

a Eta statistic.

b Arthritis, cancer, heart disease, hypertension, stroke, asthma, insulin-dependent and independent diabetes, inflammatory bowel disease, attention-deficit disorder, bipolar disorder, anxiety, and depression.

c Median = 2, range = 0–100.

*p < .05; **p < .001.

### Logistic regression models

Table 3 reports adjusted odds ratios (aORs) and associated 95% confidence intervals from binary logistic regression models examining the predictors of use of mental health services and supports. In the Model 1, analysis demonstrated significant positive associations per additional year of age [aOR, 1.16; 95% confidence interval (CI), 1.06–1.27; *p* ≤ 0.001]. In Model 2, previous mental health rating, amount of sleep during the week, and median number of school days missed were added. Of these variables, missed school (aOR, 1.85; 95% CI 1.24–2.75; *p* < 0.01) and being slightly worried (aOR, 2.03; 95% CI 1.27–3.23; *p* < 0.01) or very/extremely worried (aOR, 6.31; 95% CI 2.94–13.55; *p* *<* 0.001) in the past 2 weeks about mental health, compared with not being worried, were significantly associated with the use of mental health services and supports. In comparison, having a previous mental health rating of very good (aOR, 0.45; 95% CI, 0.28–0.73; *p* < 0.01) or excellent (aOR, 0.30; 95% CI 0.17–0.52; *p* < 0.001) was protective against MHSS use. The contribution of weekday sleep was non-significant.

**Table 3:** Hierarchical logistic regression results predicting use of mental health support(s)^a^ (*N* = 833).

Predictor variables	Model 1	Model 2	Model 3	Model 4
	aOR [95%CI]	aOR [95%CI]	aOR [95%CI]	aOR [95%CI]
Age (years)	1.16*** [1.06–1.27]	1.10 [1.00–1.22]	1.08 [0.97–1.19]	
Sex (ref = male)	1.44 [1.00–2.08]	1.13 [0.76–1.68]	1.22 [0.81–1.84]	
Income support (ref = no)	0.68 [0.44–1.03]	0.56 [0.36–0.88]	0.56* [0.35–0.89]	
School missed (ref = ≤2 days)		1.85** [1.24–2.75]	1.74** [1.16–2.61]	
Sleep weekdays (h) (ref = 8–10)				
<6		1.30 [0.29–5.79]	1.18 [0.26–5.42]	
6–8		1.58 [0.32–7.75]	1.47 [0.29–7.45]	
>10		1.06 [0.24–4.65]	0.93 [0.21–4.20]	
Previous mental health score (ref = good)				
Excellent		0.30*** [0.17–0.52]	0.33*** [0.19–0.59]	
Very good		0.45** [0.28–0.73]	0.48** [0.29–0.78]	
Fair/poor		1.25 [0.56–2.79]	1.05 [0.46–2.39]	
How worried have you been about your mental health in the last 2 weeks? (ref = not worried)				
Slightly		2.03** [1.27–3.23]	1.86* [1.15–3.00]	
Moderately		1.94 [0.98–3.85]	1.87 [0.95–3.71]	
Very/extremely		6.31*** [2.94–13.55]	5.25*** [2.38–11.56]	
Chronic health condition (ref = no)			2.78*** [1.85–4.19]	
Chronic health condition × income support				1.50 [0.58–3.84]
Chronic health condition × missed school				0.79 [0.34–1.79]
Chronic health condition × worried about MH past 2 weeks				0.78 [0.30–2.00]
Chronic health condition × previous mental health score				0.82 [0.51–1.31]

a 0 = Did not access mental health services, 1 = Did access mental health services.

* *p* < 0.05.

** *p* < 0.01.

*** *p* < 0.001.

As one of our key objectives was to examine MHSS use during COVID-19 among Canadian children and adolescents with chronic conditions, Model 3 was adjusted for previously identified confounding variables and included the addition of the chronic condition variable. After controlling for sociodemographics, previous mental health rating, number of days of missed school, and hours of sleep per night, we found that having a chronic condition was associated with MHSS use (aOR, 2.78; 95% CI, 1.85–4.19; *P* < 0.001). All of the variables remained significant in Model 3 (Table 3), with the addition of accessing COVID-19 income support (aOR, 0.56; 95% CI 0.35–0.89; *p* < 0.05). Model 4 was run to test for interactions between having a chronic health diagnosis and predictors associated with MHSS use; there were no significant interactions between predictor variables.

Finally, a subanalysis was run to differentiate those with a chronic health condition from those without (Table 4). All significant predictors were force entered to predict mental health service and support use in a chronic health model and a non-chronic model. While severe mental health worry consistently predicted service use across both groups, children without chronic health conditions showed a more complex pattern of using MHSS. In the chronic model, being very/extremely worried (aOR, 4.98; 95% CI 1.70–14.62; *p* *<* 0.01) in the past 2 weeks about mental health was the only significant predictor compared with not being worried. In comparison, the non-chronic model also had being very/extremely worried (aOR, 5.67; 95% CI 1.79–17.98; *p* *<* 0.01) in the past 2 weeks about mental health compared to not worried as a predictor, but also being slightly worried (aOR, 2.14; 95% CI 1.13–4.04; *p* *<* 0.05) and missing >2 days of school at any point during the school year (aOR, 1.99; 95% CI 1.15–3.45; *p* *<* 0.05). Not accessing COVID-19 income support was protective (aOR, 0.45; 95% CI 0.23–0.86; *p* *<* 0.05) in the non-chronic model.

**Table 4:** Subanalysis of predictors for participants with chronic health conditions using mental health support(s)^a^.

Predictor variables	Chronic model^b^		Non-chronic model^c^	
	aOR	[95% CI]	aOR	[95% CI]
Age (years)	1.07	[0.91, 1.26]	1.1	[0.96, 1.27]
Income support (no ref)	0.67	[0.34, 1.32]	0.45*	[0.23, 0.86]
School missed (≤2 days ref)	1.61	[0.87, 2.97]	1.99*	[1.15, 3.45]
Sleep weekdays (h) (8–10 h ref)				
<6	1.56	[0.15, 16.62]	0.86	[0.12, 5.95]
6–8	1.91	[0.15, 23.78]	1.11	[0.14, 8.69]
>10	1.23	[0.12, 12.74]	0.65	[0.10, 4.33]
How worried have you been about your mental health in the last 2 weeks? (not worried ref)				
Slightly worried	1.63	[0.81, 3.31]	2.14*	[1.13, 4.04]
Moderately worried	2.63	[1.00, 6.95]	1.28	[0.47, 3.50]
Very/extremely worried	4.98**	[1.70, 14.62]	5.67**	[1.79, 17.98]
Previous mental health score (good ref)				
Excellent	0.46	[0.20, 1.08]	0.38	[0.09, 1.65]
Very good	0.65	[0.32, 1.35]	0.56	[0.13, 2.36]
Fair/poor	1.62	[0.87, 2.97]	1.64	[0.41, 6.64]

a 0 = Did not access mental health services, 1 = Did access mental health services.

b *n* = 230.

c *n* = 603.

* *p* < 0.05.

** *p* < 0.01.

## Discussion

This is one of the first studies to examine factors associated with the use of MHSS among Canadian children and adolescents during the COVID-19 pandemic, with particular attention to those living with a chronic health condition. Using data collected from the CHILD Cohort COVID-19 Add-On Study (10), we identified that just under one-fifth of the predominantly school-age sample accessed MHSS between January 2021 and March 2022. Notably, beyond sociodemographic factors, being very/extremely worried about mental health in the past 2 weeks, accessing COVID-19-related income support, and having a chronic health condition were found to predict children's likelihood of using MHSS.

The COVID-19 pandemic prompted an unprecedented public health measures, resulting in social distancing (both mandated and recommended), reduced non-essential in-person medical care, and lockdown measures (i.e., travel restrictions, indoor masking, curfews, home schooling) to reduce infection in most Canadian provinces (14). A review examining the impacts of these measures on mental health across the lifespan implicated both COVID-19 illness-related stressors, such as the fear of getting sick, and secondary stressors, including social isolation and reduced access to medical care for other conditions, as potential causes of the increase in mental illness worldwide (15). During the first year of the COVID-19 pandemic, the worldwide prevalence of anxiety and depressive disorders increased by approximately 25% (16), as did overall levels of mental distress in children and youth (17, 18). In the current study, we found that being very/extremely worried about mental health in the last 2 weeks was the strongest predictor of using MHSS. Ratings of excellent or very good prepandemic mental health were found to be protective, supporting our second hypothesis. Unfortunately, we have no details as to the specific mental health concerns for which the children accessed MHSS in our study, but given that 95.5% of the participants rated their prepandemic mental health as good or better, their reasons for using MHSS may reflect new anxiety-related concerns arising from the pandemic. Prior research identified an increase in anxiety and depression during the pandemic, as well as the increased technological literacy seen in younger generations, likely making it easier for school-aged children, regardless of previous mental health status, to use MHSS during the pandemic (19, 20).

Contrary to our first hypothesis, children and adolescents in our study who were previously diagnosed or living with a chronic health condition, as per the PHAC chronic disease list, had 2.78 higher odds of using MHSS than those without a chronic health diagnosis. While we acknowledge the heterogeneity in this group, this finding is of importance, given that individuals with chronic health conditions were identified as particularly at risk of experiencing a decline in mental health during the COVID-19 pandemic (21, 22). The subanalysis findings reiterate this stance, with the only predictor of MHSS use being extremely/very worried about mental health in the last 2 weeks. This may reflect heightened social isolation during COVID-19 of children with a chronic health condition, leading to worse mental health and thus higher use of MHSS (23). Similarly, the non-chronic model had a remarkably similar effect size for being extremely/very worried about mental health in the last 2 weeks. However, children without chronic health conditions showed a more complex pattern of help-seeking, including lower-threshold worry levels, school-related indicators, and socioeconomic factors. This suggests that mental health service pathways may differ between these groups, with the chronic health condition group potentially having more direct access to healthcare or different thresholds for seeking help, while the non-chronic diagnosis group may rely more on multiple behavioral and contextual indicators to use health services.

Notably, the findings of this study offer important implications for healthcare providers. Healthcare providers should be aware of the factors that are associated with using mental health services and supports. This includes recognizing the social determinants of health, which are essential components of a child's mental health and wellbeing (24). For example, one-third of our sample accessed COVID-19 income support, yet this was associated with significantly lower odds of MHSS use in adjusted analyses (OR = 0.56). This inverse association may seem counterintuitive, but could reflect several underlying mechanisms. Importantly, the association may reflect differential patterns of healthcare access among families receiving income support or residual socioeconomic confounding not fully captured by our adjusted models (25). Economic assistance alone may therefore be insufficient to overcome the broader structural and practical barriers that limit MHSS use (26). Regardless, healthcare providers should be familiar with the full range of community-based MHSS and prepared to connect children and families with resources that align with their socioeconomic circumstances, geographic location, preferred modality, and specific health concerns. Families of children with mental health problems have been found to lack knowledge, face stigmatization, and experience service unavailability (27), so it is important to decrease barriers in accessing MHSS.

### Strengths and limitations

Our results extend previous findings from adult populations to Canadian children, showing that during the COVID-19 pandemic, MHSS use was higher among those managing chronic conditions. However, these findings are subject to some limitations. While self-reported measures are appropriate for capturing subjective experiences such as mental health worry and service use, they may introduce recall bias given the reporting window, potentially leading to underreporting of brief or infrequent service contacts. Social desirability bias may have influenced responses, as stigma surrounding mental health could discourage disclosure of service use, while proxy reporting by parents for younger children introduces a further layer of potential underreporting if parents were unaware of services their child used independently. Although we found that having a chronic condition was a predictor of using MHSS, the broad categorization of chronic conditions in our study may limit the specificity of this finding. Furthermore, as our sample was taken from the original CHILD Study, which recruited participants across four Canadian provinces and did not necessarily target children with chronic conditions, not all chronic conditions were represented in our sample. Nonetheless, our sample aligns with national data indicating that as many as 30% of school-aged children are affected by a chronic illness (28). Further research should examine MHSS use among children with chronic conditions using a sample that is representative of that population, while also exploring underlying reasons for service utilization patterns.

## Conclusion

Mental health concerns are rising among Canadian children, alongside a growing demand for timely and effective care. Identifying factors that predict MHSS use in the pediatric population can support earlier, more personalized interventions, leading to improved outcomes by promoting their mental wellbeing (29). Beyond clinical care, this knowledge can inform public health efforts aimed at prevention through education, enhancing early identification, and reducing stigma. Empowering both healthcare providers and the general public with an understanding of these predictive factors may encourage proactive engagement with mental health services, normalize help-seeking behaviors, and support a more inclusive environment for children. Integrating mental health promotion into pediatric care is essential to reducing the long-term burden of mental health conditions and may help support improved developmental outcomes as children transition into adulthood. Taken collectively, our findings contribute to the growing body of evidence identifying key predictors of MHSS use, offering valuable insights as policies are developed for equitable mental health care.

## Data Availability

The raw data supporting the conclusions of this article will be made available by the authors, without undue reservation.
