# Supplementary material for: Identifying predictors of use of mental health services and supports during the COVID-19 pandemic: an analysis of the CHILD COVID-19 Add-on Study
Source: Front Health Serv. 2026 Jun 18;6:1780564. doi: 10.3389/frhs.2026.1780564 (PMC13323234; doi:10.3389/frhs.2026.1780564)
Supplement: Supplementary file 1 [file Table1.docx]

Supplementary Table 1. Frequency count of chronic conditions^*^ represented by child cohort included in the analysis; including past or current diagnoses (N = 1,553)

| Chronic condition | Yes  n | (%) | No  n | (%) |
| --- | --- | --- | --- | --- |
| Anxiety | 189 | 12.2 | 1364 | 87.8 |
| Arthritis | 4 | 0.3 | 1549 | 99.7 |
| Asthma | 199 | 12.8 | 1354 | 87.2 |
| Attention deficit disorder (ADD) | 133 | 8.6 | 1420 | 91.4 |
| Bipolar disorder | 0 | 0 | 1553 | 100 |
| High blood pressure | 2 | 0.1 | 1551 | 99.9 |
| Cancer | 3 | 0.2 | 1550 | 99.8 |
| Depression | 33 | 2.1 | 1520 | 97.9 |
| Insulin-dependent diabetes | 3 | 0.2 | 1550 | 99.8 |
| Non-insulin dependent diabetes | 0 | 0 | 1553 | 100 |
| Epilepsy | 8 | 0.5 | 1545 | 99.5 |
| Heart disease | 3 | 0.2 | 1550 | 99.8 |
| Stroke | 0 | 0 | 1553 | 100 |
| Osteoporosis | 0 | 0 | 1553 | 100 |
| Irritable bowel disease | 1 | 0.1 | 1552 | 99.9 |

^*^ Chronic conditions defined by the Public Health Agency of Canada
